# Obstructive Sleep Apnea Hypopnea Syndrome among Obese Patients Visiting the Outpatient Department of a Tertiary Care Centre

**DOI:** 10.31729/jnma.8395

**Published:** 2024-01-31

**Authors:** Mimansa Dixit, Sachin Pawar, Saumya Saket

**Affiliations:** 1 Mahatma Gandhi Institute of Medical Sciences, Sevagram, Wardha, India; 2 Department of Physiology, Mahatma Gandhi Institute of Medical Sciences, Sevagram, Wardha, India; 3 Vellore Institute of Technology, Vellore, Tamil Nadu, India

**Keywords:** *obesity*, *obstructive sleep apnea syndrome*, *polysomnography*, *waist circumference*

## Abstract

**Introduction:**

Obstructive sleep apnea-hypopnea syndrome is a major health public problem, with a possible contribution to cardiovascular disease and obesity. There is evidence of a bidirectional relationship between obesity and obstructive sleep apnea-hypopnea syndrome. There is little to no reporting of obstructive sleep apnea-hypopnea syndrome in rural subjects. The study aimed to find out the prevalence of obstructive sleep apnea hypopnea syndrome among obese patients visiting the outpatient Department of a tertiary care centre.

**Methods:**

A descriptive cross-sectional study was conducted among obese individuals who attended a tertiary care centre between 4 June 2018 to 6 August 2018. Ethical approval was obtained from the Institutional Ethics Committee. A convenience sampling method was used. The point estimate was calculated at a 95% Confidence Interval.

**Results:**

Among 33 patients, obstructive sleep apnea-hypopnea syndrome was seen in 5 (15.15%) (2.92-27.38, 95% Confidence Interval) patients. Among 33, 3 (60%) were female and 2 (40%) male.

**Conclusions:**

The prevalence of obstructive sleep apnea-hypopnea syndrome was similar to other studies done in similar settings.

## INTRODUCTION

Obstructive sleep apnea-hypopnea syndrome (OSAHS) is characterized by recurrent episodes of apnea and hypopnea which result in successive episodes of cessation or decrease in respiratory airflow, in which obesity is an important risk factor. It is defined by an apnea-hypopnea index (AHI) of five or more episodes per hour associated with daytime somnolence.^1,2^

While obesity is one of the major risk factors for developing OSAHS, it is the only one that is reversible. The public health impact of OSAHS is now enormous in developing countries like India, with a potential contribution to the increased rates of cardiovascular disease and obesity, with a study estimating OSA prevalence of 2.6% in an Indian community-based sample.^1,2^

The study aimed to find out the prevalence of obstructive sleep apnea hypopnea syndrome among obese patients visiting the outpatient Department of a tertiary care centre.

## METHODS

This descriptive cross-sectional study was conducted among patients visiting the outpatient department of the Community Medicine department at Kasturba Hospital, Mahatma Gandhi Institute of Medical Sciences, Sevagram, Maharashtra, India. Data was collected from 4 June 2018 to 6 August 2018. Ethical approval was taken from the Institutional Ethics Committee (Reference number: MGIMS/IEC/PHY/18/2018). Participants aged 40-60 years residing in an area under primary health care, and those who gave the informed consent were included in the study. Participants having any connective tissue disorder, chronic liver disease, chronic lung disease, immunosuppressive diseases, or malignancy were excluded from the study. A convenience sampling method was used. The sample size was calculated with the following formula:


$$\begin{array}{ll} \text{n} & ={\text{Z}}^{2}\times \frac{\text{p}\times \text{q}}{{\text{e}}^{\text{2}}}\\ & =1.96^{2}\times \frac{0.02\times 0.98}{0.05^{2}}\\ & =30 \end{array}$$

Where,

n = minimum required sample size
Z = 1.96 at 95% Confidence Interval (CI)
p = prevalence taken as 2% from previous study^2^
q = 1-p
e = margin of error, 5%

The minimum required sample size was 30. However, the final sample size taken was 33.

A questionnaire on demographics, sleep symptoms, medical history, and medications was completed. Body habitus was measured in light clothing and bare feet using standard anthropometric methods. A physical examination was performed in which height, weight, body mass index, neck length, neck circumference, percentage predicted neck circumference, waist circumference, hip circumference, waist-hip ratio, and mid-arm circumference were measured according to standard methods. Polysomnography (PSG) tests were performed using the sleepcare system. Participants were categorized by apnea-hypopnea index (AHI) into mild (5-14.9), moderate (15-29.9), and severe (≥30) OSA.^3^

Data were entered and analysis was performed using IBM SPSS Statistics version 26.0. The point estimate was calculated at a 95% CI.

## RESULTS

Among 33 patients, the prevalence of OSAHS was 5 (15.15%) (2.92-27.38, 95% CI). Among them, 3 (60%) were female and 2 (40%) male. Mean height of participants was 155.50±5.90 cm (Table 1).

**Table 1:** Characteristics of participants with OSAHS (n = 5).

Parameters	Mean±SD
Age (years)	49.22±5.80
Height (cm)	155.50±5.90
Weight (kg)	83.35±9.21
BMI (kg/m^2^)	32.0±2.0
Waist circumference (cm)	107.0±9.38
Neck circumference (cm)	40.0±3.79
Apnea-hypopnea index	7.12±1.02
Epworth sleepiness score	8.72±2.23

Among them, 4 (80%) had mild OSAHS (Table 2).

**Table 2:** Distribution of OSAHS according to severity (n= 5).

Severity of OSAHS	n (%)
Mild	4 (80)
Moderate	1 (20)
Severe	-

## DISCUSSION

Among 33 obese participants, the prevalence of obstructive sleep apnea-hypopnea syndrome was 5 (15.15%). In a similar study, the prevalence of OSA (apnea-hypopnea index [AHI ≥5] in adults 30 to 69 years of age is approximately 17%, and the proportion of mild-to-moderate OSA attributable to excess weight and a BMI of >25 kg/m^2^ is 41 to 58%.^4^ In a previous study, a prevalence rate of 19.5% was seen in healthy urban Indian males aged 35-65 years, and another study reported a 13.74% prevalence rate.^2,5^

The current study shows that the prevalence of OSA is frequent in our population set up of rural Central India. In another study, OSA prevalence rates 50% in females, and its prevalence increases with age independent of other risk factors including obesity.^6^ In the current study, we have found BMI to have the most highly significant association with the occurrence of OSA. This is in accordance with the results of another study.^4,7-9^ Also observed was a significant correlation between waist circumference and the presence of OSA, comparable to the findings of the studies.^10-11^

There are some limitations to this study. This is a cross-sectional study consisting of a small sample size. This type of study design can not establish a temporal relationship of obesity with OSA.

## CONCLUSIONS

The prevalence of obstructive sleep apnea hypopnea syndrome obese patients was found to be similar to other studies conducted in similar settings.
